# A Success Story Leading Us to Think Big!

**DOI:** 10.1155/2011/215675

**Published:** 2011-11-23

**Authors:** Jasmine Kamboj, Rahul Kamboj, Tejas Shah, Pam Khosla

**Affiliations:** Department of Hematology and Oncology, Mout Sinai Medical Center, Chicago, IL, USA

## Abstract

Immune dysregulation is the hallmark of all autoimmune diseases. It is extremely interesting to study the associations and pathogenesis of the various autoimmune diseases, like the link between the AIHA and CLL. This link is well established and is based on the fact that there is loss of tolerance to the self-antigen, which in turn leads to immunebased hemolytic anemia. Around 30% of the patients with CLL are at the risk of developing AIHA, and 11% eventually develop AIHA. Whether there is any definite linkup between the corrupted immune system and “acute” leukemias/lymphomas is yet to be established. Needless to say, if there was an association between the pathogenesis of the ALLs and AIHA, it would be a landmark in the field of oncology as it would enforce early diagnosis and treatment for the disease which is much more aggressive and found in a comparatively younger age group (predominantly in children and a mean age of 40 years in adults) as compared to its chronic counterparts. The AIHA would serve as a “tip to the underlying iceberg” in these situations, warning us of the cryptic diagnosis.

## 1. Introduction

Immune dysregulation is the hallmark of all autoimmune diseases. It is extremely interesting to study the associations and pathogenesis of the various autoimmune diseases, like the link between the AIHA and CLL. This link is well established and is based on the fact that there is loss of tolerance to the self-antigen, which in turn leads to immune-based hemolytic anemia. Around 30% of the patients with CLL are at the risk of developing AIHA, and 11% eventually develop AIHA. Whether there is any definite linkup between the corrupted immune system and “acute” leukemias/lymphomas is yet to be established. Needless to say, if there was an association between the pathogenesis of the ALLs and AIHA, it would be a landmark in the field of oncology as it would enforce early diagnosis and treatment for the disease which is much more aggressive and found in a comparatively younger age group (predominantly in children and a mean age of 40 years in adults) as compared to its chronic counterparts.

 The AIHA would serve as a “tip to the underlying iceberg” in these situations, warning us of the cryptic diagnosis and so forth.

Here we present a very interesting case which is indicative of AIHA antecedent to the diagnosis of ALL.

## 2. Case

A 40-year-old Hispanic female with no significant past medical history was first seen as an outpatient by her primary care physician complaining of generalized weakness for four weeks and a 7 lb weight loss. Patient reported heavy menstrual bleeding for last 6 to 7 months.

The patient also complaint of fevers, chills, night sweats, and breathing difficulty (B symptoms). There were no complaints of any bleed, fresh or streaky, in the cough or vomitus and no complaints of any bleed per rectum, in form of tarry sticky stools or frank blood. In the social history, she belonged to an average socioeconomic status, and her eating habits were of a healthy wholesome diet. She had no history of alcoholism. She had never worked in any industrial or potential carcinogenic environment. She had no history of any NSAID or corticosteroid use and no Family history of any kind of hematological disorders or any malignancies. On physical examination she was found to have warm and moist skin with no stigmata of chronic liver disease or any subcutaneous bleed. She was afebrile, anicteric, with no pallor, no tachycardia, no lymphadenopathy, no thyromegaly, no hepatosplenomegaly, no sternal tenderness, no orthostatic hypotension, and stool occult blood negative.

Her CBC was noted to be with the hemoglobin 7.5 gm/dL (L), hematocrit 21% (L), RBC 2.4 mil/mm^3^ (L), MCV 87.4 fl, MCH 31.1 pg, MCHC 35.6%, RDW 12.6, WBC—5.4 th/mm^3^, neutrophils 39%, lymphocytes 54% (H), platelets 149 th/mm^3^, ANC 0.4 with 0.1 bands, reticulocyte count of 0.3, LDH 450 (H).

Pertinent abnormalities in the CMP were a total bilirubin of 1.3 (H), direct bilirubin 0.3 (H). The rest of the CMP, liver function tests and renal function tests, did not show any abnormalities or any electrolyte imbalance. an iron Panel, which ruled out iron deficiency anemia, was done. B12 with a value of 789 and MCV of 87.4, ruled out macrocytic anemia.Viral serologies for HIV and hepatitis were nonreactive. Chest X-ray was within normal limits.

Her anemia being symptomatic at Hb of 7.5, she was hospitalized for blood transfusion and an extensive anemia workup was done. Hemoglobin status after two units of blood transfusion was 9.2.

Hemoglobin electrophoresis showed the following: HBA—96.0/HBA2—2.9/HBF 1.1/Sickle Cell Negative. Haptoglobin was 17. Direct Coombs test was done and it was positive (IgG negative and C3 tomplement positive), and hence hemolysis was suspected. A peripheral smear showed anisocytosis with spherocytes.

 A bone Marrow biopsy was done to further define the cause, the results of which showed a precursor B lymphoblastic leukemia involving the bone marrow (51%) and peripheral blood (1%). BM biopsy showed CD34 neg, TDT pos, CD10 pos B lymphoblasts, also confirmed by flow cytometry.

Cytogenetics showed normal chromosomes 46 XX, and FISH was negative for BCR-ABL.

She got an extensive workup for staging of the leukemia, in form of CT scan of chest, pelvis, and abdomen, which was conclusive for splenomegaly but no lymphadenopathy or any other organ involvement elsewhere. She also got a MUGA scan which documented an EF of 65%.

She was started on Hyper-CVAD alternating with high dose Ara-C and methotrexate chemotherapy regimen, comprising cytoxan, doxorubicin, vincristine, and dexamethasone, with intrathecal methotrexate alternating with Ara-C. The intrathecal regimen was discontinued after two cycles of chemotherapy, after two consecutive lumbar punctures were negative for malignancy. Repeat CTs of the brain, at 3-month interval, were scheduled for 1 year, as a followup. She was given G-CSF support on need basis. Bone marrow transplantation was discussed with the patient, as she had no poor prognostic features; however the patient opted out of it owing to insurance issues.

Her pertinent labs after 6 months of therapy are Hb 13.1/Hct, 38.5/platelet, 182/RDW, 14.7/LDH 220. She did have intermittent episodes of diarrhea, *C. difficile * colitis and 1 episode of neutropenic fever, appropriately treated with antibiotics. The bone marrow showed a complete remission with less than 5% blasts, no evidence of leukemia, and normal M : E ratio. She was started on maintenance POMP thereafter.

## 3. Discussion

The association of the autoimmune diseases and lymphoproliferative disorders (mostly chronic lymphocytic leukemias and B cell non-Hodgkin lymphomas) is well known. There have been some case studies [1, 2] about the link of acute lymphocytic anemias with AIHA as well. 

Whether or not we can define a strong linkup between the acute lymphoblastic leukemia and AIHA, or AIHA developing as a complication to AIHA, or ALL developing as a complication to AIHA is one of the questions that need to be answered.

The target of such acute lymphoblastic anemias is the younger age group (children and adults with a mean age of 40), and this further stresses on the early start and aggressive approach in the treatment modality.

Needless to say, such an association, if established, will emphasize the need to recognize the underlying malignancy in the not so disastrous looking anemias.
